# A Qualitative Exploratory Study of Facilitators and Barriers Influencing Organ Donation in Ghana: Insights for Health Policy and Advocacy

**DOI:** 10.3390/ijerph23060762

**Published:** 2026-06-05

**Authors:** Kwadwo Amoah, Gladys Fordjuor, Seth Lartey, Gilbert Batieka Bonsaana, Benjamin Abaidoo, John Tetteh, Enam Afi Mana Aidam, Kwame Asante Darko, Kwame Oteng, Alhassan Fuseini, Amanda Evelyn Forgah, Mohammed Alphazaazi Quaye, Lahari Salma Abdul-Rahaman, Amina Ponaa Mamani, Dorcas Baah Boateng, Josiah Charinga, Ida Mbamah Ansing, Lawrence Oppong Kusi-Appiah, Collins Adjei Asante, Awo Yaa Karikari Oduro, Bertha Ellen Wryter, Kojo Blankson, Nafisatu Odokai Odoi, Dora Sam-Brew, Mabel Oparebea Krow, James Addy, Michael Gyamera Oboo-Gyan, Shannan Berzack, Ashiyana Nariani

**Affiliations:** 1 Eye Department, Komfo Anokye Teaching Hospital, Adum-Kumasi P.O. Box 1934, Ghana; 2 Eye Department, Cape Coast Teaching Hospital, Cape Coast P.O. Box CT 1363, Ghana; 3 Eye, Ear, Nose and Throat Department, School of Medicine and Dentistry, Kwame Nkrumah University of Science and Technology, Kumasi AK-448-4944, Ghana; 4 Eye Department, Korle Bu Teaching Hospital, Korle Bu, Accra P.O. Box KB369, Ghana; 5 Department of Ophthalmology, School of Medicine, University for Development Studies, Tamale P.O. Box TL1350, Ghana; 6 Ophthalmology Unit, Department of Surgery, University of Ghana Medical School, Accra P.O. Box 4236, Ghana; 7 Department of Community Health, University of Ghana Medical School, Accra P.O. Box 4236, Ghana; 8 Eye Unit, The Bank Hospital, Block F6, Shippi Road, Cantonments, Accra P.O. Box CT 1224, Ghana; 9 Cure Blindness Project, Ghana Office, 12 Morendo Street, Lapaz, Accra P.O. Box 863, Ghana; 10 Herbert Wertheim College of Medicine, Florida International University, 11200 SW 8th St AHC2, Miami, FL 33199, USA; 11 Cure Blindness Project, Norwich, VT 05055, USA

**Keywords:** organ donation, facilitators, barriers, motivators, transplant, organ banking, tissue banking

## Abstract

**Highlights:**

**Public health relevance—How does this work relate to a public health issue?**

- Conversations on organ donation directly address the global shortage of organs, which is a critical public health issue tied to preventable deaths and reduced quality of life.
- The findings will help in identifying cultural, social, knowledge gap, and systemic factors that influence donation rates, which are essential for improving health equity.

**Public health significance—Why is this work of significance to public health?**

- This study highlights how knowledge gaps, socio-cultural beliefs, and religious influences affect organ donation.
- Perspectives of expert health professionals are key to the provision of credible insights that can guide national strategies to improve organ donation systems.

**Public health implications—What are the key implications or messages for practitioners, policy makers and/or researchers in public health?**

- Findings may help in designing interventions for addressing the knowledge gap, cultural, and religious concerns, ultimately supporting the establishment of a deceased organ donation program in Ghana and improving access to life-saving transplants.
- Practitioners and policymakers should prioritize awareness campaigns, addressing the knowledge gap, and legislative frameworks to normalize organ donation and dispel myths.

**Abstract:**

**Background:** Organ donation and transplantation are among the greatest scientific discoveries of our time, which have restored hope and life to many. However, several factors influence the global organ donation rate. It is, therefore, important to understand the Ghanaian context of facilitators and barriers to donation to dispel cultural myths and misconceptions about organ donation. This study aims to qualitatively explore the facilitators and barriers influencing organ donation in Ghana. **Methods:** This was a qualitative exploratory study conducted among health professionals at four major tertiary hospitals in Ghana. Participants were chosen using the purposive sampling technique. Using a structured interview guide, an in-depth interview was conducted to gather qualitative data, which was then tape-recorded and transcribed. Thematic content analysis was used to manually analyze the data. **Results:** Of the 25 expert participants, the majority (15, 60.0%) were female. The majority (15, 60.0%) were between 40 and 59 years. The mean age was 42.4 ± 8.0 years. The average number of years of work experience was 15.8 ± 7.1 years. Themes identified for facilitators of organ donation included increased awareness and knowledge campaign, societal influence, and legislative support. Themes for barriers were inadequate knowledge, socio-cultural influence, religious beliefs, and ethical concerns. **Conclusions:** Increased awareness and knowledge campaigns, societal influence, and legislative support are the significant facilitators of organ donation in Ghana, whereas inadequate knowledge, socio-cultural, and religious influence are important barriers to organ donation in Ghana.

## 1. Introduction

Globally, organ and tissue failure results in a large amount of patient morbidity and mortality [1]. Although organ and tissue donations are increasing, the rising burden of reported morbidities has created a growing demand for donor organs to meet the needs of patients [1,2]. Organ and tissue donation is the act of giving one’s organs or tissues to another person in need in order to improve their quality of life or save their lives [2]. Individuals of all ages and backgrounds are eligible to donate organs and tissues [1,2,3]. Donating an organ can save many lives by reinstating vital functions in situations where there are no suitable substitutes [1,4]. Organ donation remains a process that has significantly changed the field of medical practice and provides people with key lifesaving intervention [1,4].

The estimated number of organ transplantations in 2022 globally was over 157,494 [1]. The actual deceased organ donors were reported to be 41,792 in 2022. Despite the fact that thousands of life-saving organ transplants are carried out yearly, there is still a global need for organ donors [1]. In the year 2023, Spain’s organ donation data show that the majority of donations were from deceased donors (2346), while living donations accounted for 435 cases. Globally, the distribution of organ transplants reveal that kidney transplants (39%) are the most often performed treatment, followed by liver transplants (24%) [1].

Organ donation is complex and multifaceted, encompassing legal, ethical, structural, medical, and social aspects which need to be explored [4,5,6]. Some studies, for example, have highlighted the following as both facilitators and barriers: awareness of and readiness to donate an organ; ethical dilemmas; personal spirituality and beliefs; motivation; and responsibility for the transplanted organ [5,6,7,8].

In the recent past, Ghana has made some significant strides in improving the delivery of healthcare in general. Nevertheless, there have been several reports of increasing metabolic morbidities in the country, such as kidney and liver failure, highlighting the potential demand for organ donation [9,10,11,12]. Additionally, in sub-Saharan Africa (SSA), Ghana is among the countries with the highest incidence of road traffic accidents (RTAs), often resulting in the deaths of healthy and young individuals, which represent a potential source of healthy organs for donation [13,14]. Notwithstanding, organs from these RTA victims remain unutilized as a result of barriers, which include the absence of policies and legal framework for deceased organ donation, inadequate medical infrastructure, and low public awareness about organ donation [15].

Transplantation encompasses the transfer of organs and tissues such as kidneys, livers, and corneas [1], with corneal and renal transplantation being most relevant to Ghana due to the high burden of corneal blindness and end-stage renal disease [16]. Ghana’s population of over 33 million faces limited access to transplantation, as current practices rely mainly on living kidney donation and sporadic corneal grafts from imported corneas, with no comprehensive legal framework for deceased donation [16]. Cultural beliefs and traditions strongly influence attitudes toward donation, echoing findings from other contexts, such as the UK, where cultural rather than religious barriers often hinder participation [5].

The Ghana blindness survey shows that there are about 26,000 Ghanaians who are blind from corneal diseases. Many patients are in urgent need of corneal transplantation to restore their eyesight. The majority of those who are corneal blind may never be able to see again if legislation permitting organ donation is not passed [16]. Exploring facilitators and barriers to organ donation in Ghana is a crucial step in bridging this gap between organ demand and supply and in addressing complexities tied to organ and tissue donation practices and transplantation in the country. Healthcare professionals in Ghana play a crucial role in organ and tissue donation processes as their level of knowledge, attitudes, and experiences may significantly influence practice, policy, and public trust in general. Exploring their perspectives in organ donation is therefore crucial in revealing systemic facilitators and barriers that can inform culturally sensitive policy advocacy and implementation. Understanding these barriers and facilitators will guide policy reforms, capacity-building initiatives, and awareness campaigns that align with community values, while clarifying uncertainties surrounding donor consent to support the development of a legislative framework for organ donation in Ghana [17,18].

## 2. Materials and Methods

### 2.1. Study Design

A qualitative exploratory design was used to study the facilitators and barriers influencing organ donation in Ghana. The facilitators and barriers influencing organ donation in Ghana were examined from the viewpoint of health professionals from diverse specialties. In an effort to improve transparency, we have reported our work in accordance with the Consolidated Criteria for Reporting Qualitative Research (COREQ) [19]. We chose an exploratory design because it provides a basis for understanding the research goal, allows for interaction between the researcher and study participants, and encourages participants to provide detailed information. Our guiding theory was social constructivism, which gives study participants the chance to contribute meaningfully to the topic at hand based on their unique personal and social circumstances [20].

### 2.2. Study Sites

The research was carried out in four major Ghanaian tertiary hospitals. These hospitals are the largest referral facilities in the country with specialized medical care, training and education. One of the hospitals is a national referral center with over 2000 beds, multiple clinical and diagnostic departments, and several Centers of Excellence. It records thousands of outpatient visits daily and provides advanced treatment procedures in subspecialties such as radiotherapy, nuclear medicine, neurosurgery, pediatric surgery, dentistry, ophthalmology, renal care, oncology, cardiothoracic surgery, and reconstructive plastic surgery. Another hospital, located in the middle belt of the country, is a 1500-bed facility that serves as a referral center for cases from surrounding regions and neighboring countries. It is also a major teaching hospital, supporting undergraduate and postgraduate training in medicine, dentistry, pharmacy, nursing, and allied health disciplines. The third facility has an approximately 500-bed capacity and provides specialized services to the entire northern region. It collaborates with a local university to deliver undergraduate and graduate programs in nutrition, nursing, and medicine. The fourth hospital, situated in the coastal zone, is a 450-bed referral facility staffed by over a thousand health professionals. It offers a wide range of services including general and specialized clinical care, public health, rehabilitation, surgery, obstetrics and gynecology, pediatrics, diagnostic imaging, laboratory services, and pharmaceutical care.

### 2.3. Target Population

The target population for this study was healthcare professionals from various backgrounds from the participating study centers.

### 2.4. Selection Criteria

Healthcare practitioners who were willing to give verbal informed permission and had more than five years of experience and were knowledgeable in the subject were included in this study. Health practitioners with less than five years of professional experience, as well as those unable to provide informed consent or were too ill to participate, were excluded from the study.

### 2.5. Sample Size Determination

The study by Bernard [21] provided guidance on our sample size selection, suggesting that in an exploratory study, ten to twenty knowledgeable individuals should be sufficient to identify, comprehend, and address the primary goal of discussion in any well-defined care setting to achieve data saturation. Therefore, for this study, a total of 25 healthcare professionals were recruited.

### 2.6. Sampling Strategy

The purposive sampling technique was used in selecting the four centers, as these facilities are known to have experienced health professionals with various specialties. Participants in this study were purposively sampled from diverse departments across the participating facilities to ensure broad representation of perspectives on organ donation. In our selection, we focused on participants with direct involvement in patient care and related clinical decision-making processes, ensuring that respondents were positioned to provide informed perspectives on organ donation practices and challenges in Ghana. Thus, clinicians, nurses, and other allied health professionals were involved following their expertise, experience, and relevance to the study objectives. In this direction, we included participants most likely to provide rich insights into facilitators and barriers influencing organ donation within the Ghanaian healthcare context. This study’s purpose was explained to participants at times convenient for them to be approached.

### 2.7. Data Collection Tools

The interview guide used was designed following a thorough analysis of the body of existing research on organ donation. We enlisted the expertise of three organ transplant specialists to review the interview guide, ensuring its alignment with the study’s objectives and establishing content validity. Before printing the final interview guide for use in the main study, it was pre-tested at the Eye Center of Korle Bu Teaching Hospital to allow for possible revisions. A structured summary of the final interview guide deployed is shown in Table 1 below. Trained research assistants and medical officers recorded interviews on audio recording devices.

### 2.8. Study Procedure

Ethical approval was obtained from all four participating hospitals before the commencement of data collection (IRB number: (KBTH-IRB/000148/2023, CCTHERC/EC/2024/048, TTH/R&D/SR/24/015, and KATH-IRB/AP/138/24)). The World Medical Association’s (WMA) Declaration of Helsinki was adhered to. Data collection commenced in May 2024 and was completed in September, 2024. We informed participants about the purpose, procedures, risks, and benefits of this study. Written informed consent was obtained from each participant prior to their inclusion. Participation was voluntary, and individuals retained the right to withdraw at any stage without penalty. Study participants gave explicit consent for the anonymized data and findings to be published in academic and professional outlets. We have protected identifiable information to ensure confidentiality and privacy.

Relationship and rapport building were established with participating health professionals to improve their willingness to take part in the interviews. Participants were given the study’s objectives and a rundown of the suggested study tasks. Written informed consent was obtained from participants who fulfilled the inclusion criteria. For the in-depth interviews (IDIs), appointments with chosen participants were set up at an agreed time and location. Trained medical officers and research assistants recorded the interviews using voice recorders and field notes, at each of the participating sites.

Before the interviews started, permission was obtained from the respondents to record the conversation. To break the ice, the interviewers introduced themselves and the team members before starting the interview. The interview guidelines were established and given to the participants. The interviewers clarified the purpose of the interview to the respondents, who were then given the opportunity to ask additional questions and request further explanations regarding the survey. Participants were informed that they were free to withdraw from the study at any time, for any reason, without being required to provide a justification.

Study participants provided demographic information, including gender, age, marital status, educational background, specialty, and years of experience. The interviewers asked questions about general obstacles to organ donation as well as possible facilitators.

Interviews were conducted in English and audio-taped. During the interview, important statements and information from the respondents were documented using notes. The duration of each IDI was about 45 min. After each interview, the recordings were promptly transcribed and carefully reviewed before the next one was conducted.

### 2.9. Patient or Public Contribution

In this study, there was no direct involvement of patients and the public due to the exploratory nature of the study and the reliance on interviews from healthcare personnel. While participants were not directly involved in the preparation and writing of the manuscript, their contributions were vital to the conduct and analysis of this study.

### 2.10. Data Management

The data acquired were saved on an external hard drive dedicated to the study. Data was protected using the highest quality standards and compliance with regulations and procedures established for the protection of human participants’ information [22]. Every piece of information gathered was written and secured with a password that the investigators alone could access. Respondents’ identities were withheld; instead, participant responses were identified by codes. To guarantee that the transcription process was accurate, we anonymized each transcript and used defined procedures to control and correct it. Before the data analysis, an independent investigator was tasked to verify that the data had been correctly transcribed and anonymized.

### 2.11. Reflexivity and Rigor

The study design, methodology, results, and conclusions were all well documented in the manuscript to ensure reflexivity and rigor and to increase the outcome’s credibility. Credibility, dependability, conformability, and transferability were used in this study [23,24]. By employing suitable techniques for data collection and analysis and by enlisting the help of all research authors to jointly examine and offer feedback on the transcripts, we were able to ensure the study’s authenticity. The data analysis strategy illustrates the stability and consistency of the data acquired, and dependability was guaranteed by providing a clear explanation of the technique used in the data gathering.

Confirmability was achieved by audio recording and verbatim transcription of the interviews, followed by a field note check by an independent researcher to verify the accuracy of the transcription. We ensured transferability by giving readers enough information about the data collected, including the sociodemographic traits of the respondents, to allow them to assess how applicable the data is in different contexts. To increase openness, we also adhered to the COREQ standard.

### 2.12. Data Analysis

Data analysis was done manually. Microsoft Excel 2015 was used to record and evaluate demographic information such as gender, age, marital status, educational background, job status of respondents, and years of experience. Descriptive data was reported as means and standard deviation. Categorical data was presented in percentages and counts. Codes were developed and organized into themes. Thematic content analysis was used in compiling all relevant information for each possible theme. We evaluated the themes’ applicability to the coded extracts, looked for more themes, and improved the themes by giving each one precise names and definitions.

## 3. Results

### 3.1. Demographic Profile of the Health Professionals in the Study

#### 3.1.1. Sex and Age Distribution

- A total of 25 health professionals participated in this study. Most (15, 60.0%) of the health professionals were females.
- The majority (15, 60.0%) were between 40 and 59 years (middle-aged adult). The mean age was 42.4 ± 8.0 years.
- The minimum and maximum ages were 30 years and 58 years, respectively. The average work experience was 15.8 ± 7.1 years. The minimum work experience was 5 years, while the maximum was 30 years.

#### 3.1.2. Professionals

- Ophthalmologists constituted the largest group among the health professionals, accounting for 6 (24.0%). Twenty-one (21, 84.0%) were married, and the majority (22, 88.0%) were Christians (Table 2).

### 3.2. Facilitators of Organ Donation

**Figure 1:**
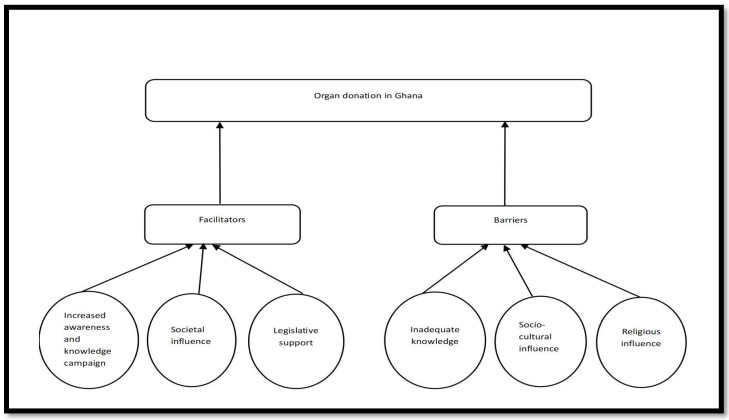
Main themes generated from the interviews.

From the interviews conducted, themes identified for facilitators of organ donation included increased awareness and knowledge campaign, societal influence, and legislative support (Figure 1).

#### 3.2.1. Increased Awareness and Knowledge Campaign

Increased awareness and knowledge campaign for organ donation creates an atmosphere for educating the public about the importance of organ donation to dispel myths and encourage participation in registration for donation. This can be accomplished by using a variety of strategies and tactics, including social media, events, gatherings with stakeholders, and meetings with target group representatives. The perspectives of health professionals on the increased awareness and knowledge campaign were as follows:

*“… creation of awareness will be one of the biggest things that we have to look at if we want to be successful with organ donation.”*—D1.

*“So firstly, I think some level of public education will go a long way in sensitizing people to understand the role that organ donation plays in improving the health and quality of lives of those living. I think health personnel should create awareness for people to know that there is an on-going program or campaign about organ donation. It will also help them to give more effective referrals and also direct patients to the right places for donation.”*—A2.

*“If there is good education on the topic and everybody understands what it involves, it is for our benefit. In the past, before blood donation became a big thing, people were skeptical about receiving another person’s blood and all that. But people realized that indeed it saves life, it has caused the people to be more open to it. So, education is important.”*—C2.

#### 3.2.2. Societal Influence

The theme “societal influence on organ donation” describes the multitude of social, cultural, and environmental elements that mold attitudes, convictions, and actions toward organ donation. Witnessing friends and family sign up as organ donors inspires people to make the commitment to donate their organs. These can also be made easier by using celebrity endorsements, in which well-known people serve as spokespersons for organ donation. The following are narrations describing societal influence as a facilitator for organ donation:

*“People may be influenced when they hear of success stories from recipients of donated organs. This may influence others to participate in organ donation.”*—D4.

*“When friends and loved ones pledge to donate their organs, it encourages people in their social circle to also pledge to donate their organs.”*—A4.

*“Well-known people in society can positively influence organ donation when they get themselves involved in organ donation by their followers.”*—C3.

#### 3.2.3. Legislature Support

Promoting organ donation could be made easier by legislative support for the practice. Depending on the reason for organ and tissue donation, different laws and restrictions may be required. Different jurisdictions may have different standards for consent; some use opt-in, or explicit consent, while others may subscribe to opt-out. Whichever structure is employed, there must be clarity regarding the legal requirements for declaring a desire or refusal to donate an organ. The legal underpinnings of organ donation may cover topics including informed consent for organ donation, eligibility for donation, allocation of organs and tissues to recipients, and the operations of donation and transplant systems.

In order to encourage organ donation, participants offered a baseline of legal proposals that addressed living donation, tissue donation, donation methods, and structure through the following narratives.

*“Currently, there is no law backing the process in Ghana. But then there are specialists who are knowledgeable in doing organ transplants. So having the specialists on the ground is a plus. But the equipment may not be available at the moment and the law is not there.”*—D1.

*“Ok. So, for the facilitators, the main thing we need to do is to get the government approval, government backing. So of course, if there is a law passed, it is known by the public which is being advertised by people like the churches, the mosques, opinion leaders if they get involved, they can talk to people and they will actually accept it. It will be easier for people to accept that it can be done.”*—A4.

*“Yes, because from the beginning I spoke about law being enacted for it. The passing of the law makes it easy. When they bring the body and family members are told about the procedures because a law has been passed it wouldn’t create any panic for them.”*—B3.

*“So, first of all, we don’t have any policy in Ghana concerning organ donation so that needs to be established. We must have the right infrastructure. We need trained personnel who will be able to carry it out successfully. We also need to strengthen our labs to be able to provide supportive care. Before a transplant is performed, a matching test must be carried out. Currently, we are not doing it in our labs, so that is where to start from. Getting the right infrastructure, getting people, and getting a national policy on organ donation in Ghana will be helpfull.”*—C7.

### 3.3. Barriers to Organ Donation

Themes for the barriers were inadequate knowledge, socio-cultural influence, and religious influence or concerns (Figure 1).

#### 3.3.1. Inadequate Knowledge

Insufficient knowledge about organ donation might cause misunderstandings, anxieties, and reluctance to provide an organ or tissue. These challenges stem from inadequate awareness of the benefits of organ donation, the range of organs and tissues eligible for donation, and the processes required for successful transplantation. Myths have been spread about organ donation, which include the belief that it disfigures the donor’s body, organs are sold for profit, and donations cause funeral plans to be delayed.

*“Firstly, I think the lack of knowledge is a barrier because what most people know is that right now chronic disease is one of the things that is on the rise. Probably the public has come to know that if your kidneys fail, you may be able to have a transplant, and the predominant knowledge is that if you go to India, they will do the organ transplant for you. That’s what most people know, but the knowledge that it extends to various other organs in the body may not be known so that could be one of the barriers.”*—A2.

*“Lack of knowledge. I think that is what I see most because people don’t know. Some may even think you are harvesting these organs for a ritual, so it is incumbent on us to really educate people. Once they are educated and see the benefits of this, a lot of people will accept, I think.”*—1C.

*“One of them is education for the family members or potential donors to understand the need for organ donation, to understand why the treatment is important. Also, the health workers should be educated to know that this is a potential form of treatment to their patients so that they can encourage it. So, it is not only patients and relatives but also health workers need to understand the importance of organ donation.”*—C6.

*“So, these barriers, first of all have to be overcome by education, mass education. The concept should be broken down to the very best minimum for all to understand and for all to also accept.”*—A5.

#### 3.3.2. Socio-Cultural Influence

Socio-cultural influence may negatively impact attitudes and decisions to donate organs. Organ or tissue donation may be hampered by cultural conventions and traditional beliefs about dying and maintaining bodily integrity. The lack of well-organized organ donation mechanisms in some nations makes it challenging for organ donation to be accepted generally. The following are narrations describing socio-cultural influence.

*“In Ghana, I think our beliefs is the topmost barrier. People think that when you are taking an organ for a recipient, whatever negative thing the person has will be transferred to them. It is going to be surgical procedure, and majority of our surgeries are not being covered by the insurance.”*—A4.

*“Our culture is part of the barriers. With the culture, some people don’t believe in such practices. Some of the religious groups, sorry to say, don’t believe in such things. For instance, when someone needs blood, some religions don’t accept blood from other people. Those things can be barriers. Another thing is ethical constraints.”*—B1.

*“There are many cultures and religions in this country that will frown on organ donation. Even though we know very well about the health benefits, some religious groups will say no, in my opinion. We know several people who have lost their lives because they refused to accept a blood donation, for example. Some people will say, I will rather take this to the grave. They believe in the afterlife. They want to take their eyes, their heart or their kidney to the afterlife. And all these come down to cultural and religious beliefs.”*—C2.

*“Culturally also, you know, for example, if you are taking an organ from, let’s say, a cadaver, most cultural people won’t like it because they want to have their deceased relatives whole or complete. Maybe, if they were born with two kidneys, they should die with the two; if they were born with two eyes, they should die with two eyes, and they should be buried with two eyes, and so on. So, for culture as a barrier, I think that the education will have to go far to help them to understand that doing organ donation is something that can be seen as medical good will and it will take nothing away from a cadaver that has donated organs. So, for the religious views on organ donation, I can really think of certain religions that will not condone to donating.”*—A2.

#### 3.3.3. Religious Influence

Certain religious organizations may restrict organ donation. The extent to which the beliefs of religious leaders will impact implied consent and organ donation impact or mirror those of lay followers. According to this study, participants’ religion may influence organ donation as follows:

*“Some people will say their religion does not permit the practice of donating their body parts to others. For example, the Jehovah’s Witnesses do not donate blood or accept blood from someone. Also, fear will make some people not accept donation of a body part from the dead person because they don’t have much knowledge about it.”*—B3.

*“So, usually, our belief systems, in one way or the other, is linked to our cultural values. So if you are in a type of religion that promotes organ donation or that talks about the positive impact of organ donation of course people will have certain mind-set towards that and if you are in a religion whereby I cited an example that after death there is life to live somewhere, so I don’t think those people who believe in that religion or value will be easily swayed as far as organ donation is concerned.”*—C5.

*“With religions, the traditional religion again is very tied to our culture. I wouldn’t want to pinpoint them; Ghana is cosmopolitan in terms of religion, and there are many other beliefs. Many other people have few issues with some of this.”*—A5.

*“The religious leaders will talk highly against taking a part or all your organ out of you. Most of them believe that all organs must remain intact in the afterlife.”*—D4.

## 4. Discussion

Our findings demonstrate that organ donation in Ghana is influenced by a complex interplay of facilitators, which includes increased awareness, societal influence, and legislative support, and barriers such as inadequate knowledge, socio-cultural influence, and religious influence. In this study, our novel finding is the strong interplay between cultural perceptions and legislative gaps, which uniquely positions Ghana within the broader global discourse on organ donation. Organ and tissue donation with transplant services have predominantly been familiar in advanced economies of the world [1,2]. The most apparent reason is that it is an endeavor that is characterized by many barriers, among which socioeconomic factors tend to play significant roles [1].

Organ donation is a very sensitive issue, and the decision for one, either in life or after death, can be a very weighty one. Increased awareness campaigns and knowledge among both health professionals and the lay public are critical interventions for facilitating the willingness to donate an organ. In Ghana, limited public understanding has historically hindered donation, but recent initiatives by health institutions and media outlets have begun to shift perceptions [25,26,27]. Knowledge is significant in empowering potential donors in informed decision-making, allaying myths related to the dignity of the human body after death, and underscoring the life-saving benefits of donation. In the absence of adequate public education, myths about organ donation are likely to thrive and negatively impact any drive for donation. Our respondents suggested that knowledge and awareness creation programs should be designed to target health professionals and the public in general. They also emphasized that awareness creation and knowledge dissemination campaigns should focus on the benefits of organ donation in saving and improving the quality of lives. In a similar study in India, Saxena et al. discovered that family members’ belief that organ donation would help save the lives of others was a significant motivator for donation [28]. Shaheen and Souqiyyeh also reported improved awareness creation and knowledge in the medical community and the general public as some of the key strategies that ensured the success of the Saudi Centre of Organ Transplantation (SCOT) [29].

Another important facilitator of organ donation from the study is societal influence. Societal norms and peer influence usually play a vital role in shaping attitudes. In communal-centered societies, including Ghana, decisions about health and death extend beyond the individual. Such decisions are regarded as shared responsibility, usually involving parents, the extended family, elders, and leaders of the community, and are therefore influenced by collective traditions and expectations. This supports the belief that the individual’s body and life are deeply interconnected with the family and society. Studies from sub-Saharan Africa have highlighted the significance of endorsements and influences from trusted figures such as chiefs, elders, religious leaders, and healthcare providers as key facilitators for organ donation [30,31].

Humans are fundamentally social beings, and social influences play a pivotal role in their decision-making [32]. Many individuals, left alone, would eagerly receive or donate an organ if convinced of the benefits. However, some social norms may restrict their freedom to do this. Because of this, successful organ and tissue donation programs have consistently recognized the critical role of societal influence and strategically employed it to achieve landmark targets [33,34,35]. Our respondents were very apt in acknowledging that an individual’s decision to donate an organ/tissue could be influenced by people in one’s social circles, like friends, peers, co-workers, leaders, and popular members of society. Not surprisingly, Wongboonsin et al. reported that neighborhood socioeconomic status was a key driver for people joining the organ donation program in Thailand and that an individual was more willing to be an organ donor when having neighbors with higher socioeconomic statuses [32]. Irvin et al. also identified relational ties and family influences among the factors that influenced one’s decision to be an organ donor in their systematic review of qualitative studies that explored community attitudes towards living and deceased solid organ donation [36]. These support the opinions expressed by respondents, and in Ghana, we can examine the potential of using prominent and/or celebrity figures and opinion leaders to achieve similar effects [37]. Our findings provide a unique insight into how society-driven strategies may help in overcoming barriers to enhance donor registration in global health initiatives to increase equitable access to organ transplantation in general. All the interviewees expressed the need for the law to regulate organ/tissue donations in Ghana. In organized organ donation systems, legislation plays a key role in their effectiveness. Currently, there is no comprehensive legislative framework to regulate organ harvesting and transplantation in Ghana. Several advocacy campaigns have called for parliamentary action for a legal instrument that will ensure ethical practices, protect donors, and build public trust. Our respondents explained that an enacted law on organ donation in Ghana would be an indication of government backing, which could effectively address citizens’ concerns about organ recovery and use of donated organs. Others opined that the practice of organ donation and transplantation needs to be regulated by a government policy with legal backing to address most of the potential barriers. This is exactly in tandem with the strong acknowledgement by Symeou et al. that despite the worrying global deficit in supply of organs for securing lives, ‘successful transplantation programs require strict adherence to well-defined conditions, supported by structured programs providing administrative and legislative guidance’ [38]. The international consensus forum also recommended that the organ donation architecture should have an ‘established, detailed legislation, guidance, information, and support system to clarify the donation pathway and actions required at each stage.’ [39]. Globally, countries with robust legislation have higher donation rates, underscoring the importance of legislative support in organ donation.

Although progress in the organ donation campaign has been made, inadequate knowledge remains a gap. While our study did not measure knowledge directly among the respondents, their perspectives highlight knowledge gaps as an important area for future research and intervention. Many Ghanaians are unaware of the organs that can be donated and the procedures involved [25,26,27]. This gap may promote fear and reluctance. The interviewees acknowledged the grave consequences that inadequate knowledge of the benefits of organ donation can have on any conceived project: the growth and spread of myths, rumors about the trading of organs, and ignorance about the scope of transplantable organs/tissues, leading to the trivialization of their need. Apart from health professionals, transplant patients, and perhaps their relatives, the bigger segment of the population lacks significant knowledge about organ transplant and donation. In Bangladesh, Moonajilin et al. reported that only 35.8% of respondents drawn from the adult population knew about organ donation, though 63.7% had a positive attitude towards it [40]. In their bid to explore the knowledge, attitude, and the effect of social media campaigns on willingness to donate in Eastern Saudi Arabia, Alessa et al. found that the majority of the included participants (63%) were not willing to donate their organs due to inadequate knowledge [41]. Though they found a potential positive social media effect in changing the decision to donate, fear of surgery and loss of life in the process of donating organs were found to be significant deterring factors stemming from inadequate knowledge. In Ghana, an effective information dissemination strategy must be fashioned to reach the greater proportion of the population with the right knowledge about organ donation.

Another significant barrier to organ donation from the study is socio-cultural influence. Cultural ideologies and beliefs concerning the integrity of the human body after death, coupled with ancestral traditions, may not encourage organ donation. In Ghana, some traditions believe that the whole body must remain intact for the afterlife. These traditions do not foster donation even in situations where people appreciate the medical benefits and the need for organ donation. Among the Akans in our Ghanaian society, the concept of death is seen as a transition into the ancestral world, with the human body as the channel for transporting an individual to this new world [42], hence the need for the body to remain whole to ensure safe passage and acceptance among the ancestors upon arrival. This is also evident among the Gas, who also emphasize the need to treat the human body with dignity and respect, as mishandling of the body could disrupt the spiritual transition of the dead [42]. At funeral grounds, the body is usually displayed for public viewing, reinforcing the significance of the intactness of the body as a mark of continuity between the living and the dead. Since the intactness of the body represents respect, wholeness, dignity, and readiness for the afterlife, the removal or absence of any part of the body is seen as dishonor to the deceased and a hindrance to the transitional journey to the ancestral world. Among the Yorubas and Igbos in Nigeria, and the Zulus in South Africa, traditions also emphasize respect for the dead but differ in focus [43]. For example, the Yorubas prioritize spiritual rites and offerings to the ancestors over the intactness of the human body after death, whereas the Igbos stress burial in ancestral land as essential for spiritual continuity. The Zulus also highlight ritual cleansing and ancestor communication, valuing the body yet focusing on practices that connect the living with the dead in general. This demonstrates how Ghanaian traditions emphasize the significance of bodily intactness or wholeness as a prerequisite for afterlife acceptance, whereas other African traditions emphasize the location of burial, ritual offerings, and rites [43].

Irving et al. identified cultural influences as one of the eight factors that would influence donors’ decisions, in a systematic review of eight qualitative studies [36]. Li et al. also found low organ donation registration among Chinese and Korean Americans due to cultural avoidance of the discussion of death-related topics and the desire for an intact body, mainly stemming from the Confucian concept of filial piety, in a systematic review [44]. Al-Qerem et al., on the other hand, found a few respondents reporting religious or cultural beliefs as the reason for which they would not donate an organ, in a survey involving Jordanian adults [45]. Another important barrier to organ donation is religious influence. Religion is very key in transforming individuals’ attitudes and may shape donation practices in several ways. Although neither the Holy Bible nor the Quran clearly talks about organ donation, African traditional religion emphasizes the afterlife and ancestral continuity and usually requires consultation with elders or religious leaders before health or death-related decisions are made in the community. The published literature is replete with studies that have sought to establish or refute this perception. The concerns expressed by interviewees in this study are not different. It is heartwarming to note, however, that in some regions of the globe, where one would easily conclude that religion would hamper decisions for organ donation, studies have reported otherwise. Irving et al. identified religious beliefs as one of the factors that influence one’s decision to be an organ donor (in a systematic review of qualitative studies) and indicated that it was among the seemingly intractable factors that could disrupt the donation decision-making process [36]. Doerry et al., however, reported that the umbrella organizations of five major religions (Christians, Muslims, Jews, Hindus, and Buddhists) they involved in their survey in Germany had a positive view on organ donation, particularly deceased donations. They saw the decision to donate an organ as a sign of altruism, love, and respect for another human being. In Bangladesh, despite a significant positive attitude towards organ donation, a cross-sectional exploratory study among adults indicated religious barriers as one of the four identified. All these are pointers to the potential challenges religious and socio-cultural influence can pose to any organ/tissue donation program in Ghana, and policy formulation will have to give critical attention to them. Although Ghana does not yet have an established deceased organ donation system, families are occasionally approached for organ donation, particularly kidneys. This makes socio-cultural resistance and religious beliefs relevant considerations in understanding barriers to donation. Nevertheless, we are cautious in presenting such conclusions, as the evidence supporting this assertion is limited. Future studies should therefore investigate these socio-cultural influences more systematically to strengthen the evidence base.

Beyond socio-cultural and awareness barriers, other evidences underscore the importance of organizational structures and professionalization in organ donation systems. For example, Johnston-Webber et al. highlight transferable paradigms from high-performing countries, emphasizing governance, infrastructure, and workforce training [46]. Similarly, lessons from the Spanish model demonstrate that legislative frameworks, clinical leadership, and logistics networks are central to sustaining high donation rates [47]. These insights suggest that Ghana’s future strategies must balance awareness initiatives with systemic reforms to ensure effectiveness.

Building on the barriers and facilitators of organ donation outlined by our respondents, the following recommendations are proposed to address challenges while strengthening supportive factors for improved donation practices. For example, addressing the knowledge gaps and enacting legislation could increase donor pools and reduce death from organ failure. Increased access to donated organs and transplantation services would reduce over-reliance on costly long-term treatments such as dialysis among persons with chronic kidney diseases. Organized donation campaigns in schools and religious gatherings could improve donations. There is a need to equip health professionals with accurate information to assist them in counseling their patients effectively. Engagement of religious and traditional leaders is important in aligning donation practices with cultural values.

Further research could explore the long-term impacts of awareness campaigns and the role of religious leaders in shifting norms to foster organ donation. We also recommend a comparative study across African countries to examine best practices for scaling organ donation systems. Lessons from Ghana highlight the importance of knowledge, legislative frameworks, and religious and cultural sensitivity as key issues in the quest to promote organ donation and contribute to global health goals in general. While our findings emphasized public awareness as a major barrier to organ donation in Ghana, we recognize that awareness alone cannot ensure system effectiveness. Extensive studies demonstrate that successful organ donation programs depend primarily on organizational structures and the professionalization of donation processes, particularly among critical care teams. In Ghana, where a deceased organ donation program is not yet established, both public awareness and institutional readiness are essential. Future research should therefore explore how organizational frameworks, critical care team training, and professionalization can complement awareness initiatives to create a sustainable and effective donation system.

Although our findings cannot be generalized across the entire population of Ghana, they provide valuable insights in understanding organ donation in Ghana as the study was conducted among health professionals at the four main teaching hospitals in the country, which represents a critical hub of medical expertise and practice, making the results an important contribution to understanding the facilitators and barriers to organ donation within the Ghanaian healthcare system. It is important to note that all the health professionals in this study are key stakeholders in patient care and health policy. As a result, their perspectives are valuable for understanding systemic facilitators and barriers, since organ donation requires multidisciplinary collaboration across specialties, institutional structures, and public health frameworks. Their inclusion ensures broader representation of healthcare voices relevant to organ donation discourse in Ghana.

This study is limited by its reliance on healthcare professionals’ perceptions. While insightful, it excludes medical knowledge (e.g., brain death determination, donor maintenance, and family approach), personal attitudes, and organizational structures. In Ghana, where a deceased organ donation program is not yet established, future research should broaden the scope to include these dimensions for a more comprehensive understanding of organ donation challenges. Although, this study did not examine healthcare professionals’ knowledge gaps, nevertheless, competencies in brain death diagnosis, donor maintenance, and family approach/consent are critical. We acknowledge this omission and recommend future research systematically addressing these gaps to strengthen organ donation systems in Ghana. The predominance of ophthalmologists may indicate enrolment bias as the data collection was done mainly by eyecare personnel, and this could be the reason. While ophthalmologists are not typically involved in deceased organ donation processes compared to ICU, anesthesia, emergency, neurology, or neurosurgery specialists, their inclusion is justified as corneal transplantation remains one of the most common organ donation practices globally, and ophthalmologists play a central role in this process. Furthermore, we are motivated to use these findings to support the establishment of a legal framework for corneal donation, alongside broader organ donation initiatives, in Ghana. This perspective underscores the relevance of ophthalmologists as stakeholders in shaping both clinical practice and policy development.

## 5. Conclusions

In conclusion, increased awareness and knowledge campaigns, societal influence, and legislative support serve as facilitators of organ donation in Ghana, whereas inadequate knowledge, socio-cultural, and religious influence represent important barriers to organ donation in Ghana.

## Figures and Tables

**Table 1:** Structured summary of the interview guide.

Section	Item
A	Sociodemographic characteristics of the key stakeholders
B	Facilitators of organ donation
C	Barriers to organ donation
D	Legal aspects of organ donation

**Table 2:** Demographic characteristics of the health professionals.

Demographic Characteristics	Number	Percentage (%)
Age:		
<40 years (young adults)	10	40.0
40–59 years (middle-aged adult)	15	60.0
Sex:		
Male	10	40.0
Female	15	60.0
Total	25	100.0
Number of years of experience		
5–10 years	7	28.0
>10 years	18	72.0
Center:		
A	5	20.0
B	6	24.0
C	7	28.0
D	7	28.0
Professionals:		
Anesthesiologist	1	4.0
Nephrologist	1	4.0
Cardiologist	2	8.0
Optometrist	2	8.0
Hematologist	2	8.0
Pediatrician	2	8.0
Medical Officer	4	16.0
Nursing Officer	5	20.0
Ophthalmologist	6	24.0
Marital status		
Widowed	1	4.0
Single	3	12.0
Married	21	84.0
Religion		
Islam	3	12.0
Christian	22	88.0

Mean age = 42.4 ± 8.0 years. Minimum and maximum ages = 30 and 58 years, respectively. Mean years of experience = 15.8 ± 7.1 years. Minimum and maximum years of experience = 5 years and 30 years, respectively.

## Data Availability

Data is available from the corresponding author upon reasonable request.

## Abbreviations

The following abbreviations are used in this manuscript:

SSA	Multidisciplinary sub-Saharan Africa
RTA	Road Traffic Accidents
COREQ	Consolidated Criteria for Reporting Qualitative Research
SCOT	Linear Saudi Centre of Organ Transplantation
